# SELF-RATED HEALTH AS A PREDICTOR OF DEPRESSION AND ANXIETY IN OLDER ADULT INMATES

**DOI:** 10.1093/geroni/igz038.593

**Published:** 2019-11-08

**Authors:** Carlyn E Vogel, Lisa C Barry

**Affiliations:** 1 University of South Florida, Tampa, Florida, United States; 2 University of Connecticut School of Medicine, Farmington, Connecticut, United States

## Abstract

Inmates age ≥50 years (older inmates) are a rapidly growing population within the U.S. correctional system with the highest suicide rate among adult prisoners. Although depression and anxiety are strong precursors of subsequent suicide, little is known regarding factors associated with these outcomes in older inmates. To inform suicide prevention efforts in this high-risk population, we evaluated the role of older inmates’ self-rated health (SRH) in relation to depression and anxiety. We utilized data from the ongoing Aging Inmates Suicidal Ideation and Depression study (Aging INSIDE). Participants (N=175) included men age ≥50 (M=56.5, SD=6.3, range=50-79 years) from eight correctional facilities in Connecticut who completed face-to-face interviews. The outcomes, depression and anxiety, were assessed using the PHQ-9 (range 0-27) and GAD-7 (range 0-21); higher scores on each scale indicated worsening severity. SRH, operationalized as a pseudo-continuous variable (1=excellent; 5=Poor), was correlated with depression (r=0.379; p <.001) and anxiety (r=0.260; p =.001) in unadjusted analyses. Two linear regression models were conducted to determine if SRH was associated with depression and/or anxiety after controlling for age, race (white versus non-white), years of education, visitors (yes versus no), and number of chronic conditions. Increasingly worse SRH was significantly associated with more depressive symptoms (β=1.92, SE=.43, p <.001) and higher anxiety scores (β=1.41, SE=.41, p=.001). SRH explained 10.0% and 6.2% of the variance in depression and anxiety scores, respectively. SRH may be useful for identifying older inmates who are more likely to have depression or anxiety, and thus may be at higher risk for suicide.

